# ICE-MoCha: Intelligent Crowd Engineering using Mobility Characterization and Analytics

**DOI:** 10.3390/s19051025

**Published:** 2019-02-28

**Authors:** Abdoh Jabbari, Khalid J. Almalki, Baek-Young Choi, Sejun Song

**Affiliations:** 1 School of Computing and Engineering, University of Missouri-Kansas City, Kansas City, MO 64110, USA; kjaf3f@mail.umkc.edu (K.J.A.); choiby@umkc.edu (B.-Y.C.); sjsong@umkc.edu (S.S.); 2 College of Computer Science and Information Technology, Jazan University, Jazan 45142, Saudi Arabia

**Keywords:** crowd safety management, Bluetooth low energy (BLE), Internet of Things (IoT), RSSI, mobility

## Abstract

Human injuries and casualties at entertaining, religious, or political crowd events often occur due to the lack of proper crowd safety management. For instance, for a large scale moving crowd, a minor accident can create a panic for the people to start stampede. Although many smart video surveillance tools, inspired by the recent advanced artificial intelligence (AI) technology and machine learning (ML) algorithms, enable object detection and identification, it is still challenging to predict the crowd mobility in real-time for preventing potential disasters. In this paper, we propose an intelligent crowd engineering platform using mobility characterization and analytics named ICE-MoCha. ICE-MoCha is to assist safety management for mobile crowd events by predicting and thus helping to prevent potential disasters through real-time radio frequency (RF) data characterization and analysis. The existing video surveillance based approaches lack scalability thus have limitations in its capability for wide open areas of crowd events. Via effectively integrating RF signal analysis, our approach can enhance safety management for mobile crowd. We particularly tackle the problems of identification, speed, and direction detection for the mobile group, among various crowd mobility characteristics. We then apply those group semantics to track the crowd status and predict any potential accidents and disasters. Taking the advantages of power-efficiency, cost-effectiveness, and ubiquitous availability, we specifically use and analyze a Bluetooth low energy (BLE) signal. We have conducted experiments of ICE-MoCha in a real crowd event as well as controlled indoor and outdoor lab environments. The results show the feasibility of ICE-MoCha detecting the mobile crowd characteristics in real-time, indicating it can effectively help the crowd management tasks to avoid potential crowd movement related incidents.

## 1. Introduction

**Figure 1:**
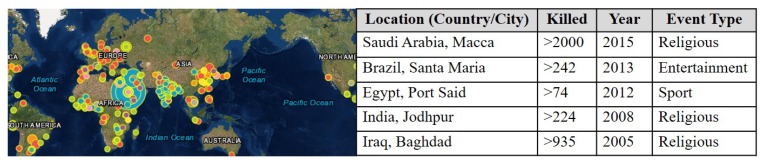
Crowd caused disasters around the global [5].

Due to the unprecedented scale and speed of urbanization, cities are facing the daunting task of accommodating the urban dynamics. The concept of smart cities attracts city planners and researchers as it facilitates many smart community services by combining cyber-physical systems and social entities through the wireless, mobile, and intelligent information and communication technologies (ICT). One of the critical service requirements of future cities is the safety management for citizens and communities [1]. Specifically, the safety management during the densely populated events such as religious, entertainment (such as sport and music), and political gatherings becomes more significant as it happens more frequently and in large scales in modern cities. Unlike static crowd events where a crowd is formed in a specific location, or when a crowd is moving from a location to another (i.e., unidirectional), it requires more space (i.e., less density). If crowd mobility exhibits multiple non-unidirectional patterns, it would require even more space to be safe and is much harder to manage or control [2,3] them. Risks of human casualties at mobile crowd events are more likely to occur because small incidents at the crowd are enough to cause panic to the attendees to start hustling, collapsing, trampling, and stampeding each other. Any inappropriate crowd management often results in disastrous repercussions such as injuries and casualties [4]. Figure 1 shows many recent cases of crowd disasters that cause human losses around the world. The community stakeholders need to provide their best efforts to maintain the crowds properly.

**Figure 2:**
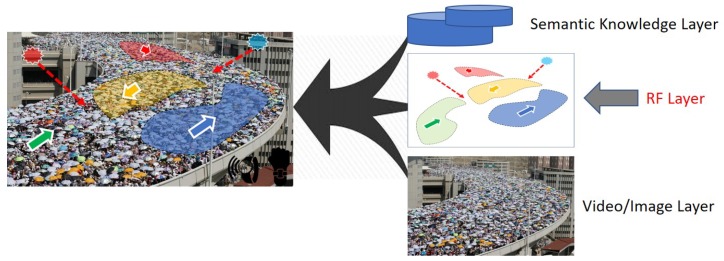
Intelligent crowd engineering architecture.

The recent smart video surveillance inspired by the advanced artificial intelligence (AI) technologies and machine learning (ML) algorithms enables a broad spectrum of promising safety applications, including object detection and identification, behavior recognition and tracking, and anomalous event detection [6,7]. However, video surveillance alone cannot identify and predict particular crowd status. It cannot scale and lacks the capacity for providing an appropriate mobile crowd safety management in real-time. For example, in Mecca, Saudi Arabia, during Hajj season, groups of pilgrims were taking the opposite road direction to get to their destination faster. When the crowd flow got clogged from the crowd flow from the opposite direction, it resulted in more than 2000 casualties. Although there were 5000 video surveillance points installed all around Mecca to monitor the Hajj season [1], the accident was not able to be prevented in time. The image layer in Figure 2 shows a very high-density crowd that is located on a bridge. However, it does not reveal the group identity and location within the crowd and their moving direction and speed.

In this paper, we propose an intelligent crowd engineering platform using mobility characterization and analytics (ICE-MoCha). ICE-MoCha enhances safety management for a mobile crowd events by predicting and preventing potential disasters through real-time radio frequency (RF) data characterization and analytics. The motivation is to improve the safety management method for the mobile crowd by filling up the scalability and capability gaps of the existing video surveillance via tightly integrating RF signal analytics (Figure 2). It implements a wireless-based, efficient and a scalable crowd/group tracking technology. Specifically, we exploit a tracking bracelet and monitoring infrastructure as well as a couple of abnormality scenarios and prediction algorithms. ICE-MoCha uses a Bluetooth low energy (BLE) [8] communication in this project as it is power-efficient, cost-effective, and ubiquitous [9]. Among the many crowd mobility characteristics, by using passive BLE scanners, we measure the number of beacons, the radio strength signals received (RSSI) value, and its variation pattern. Integrating the received beacon values, ICE-MoCha can identify the crowd density, the object group location, and the flow direction and speed. ICE-MoCha applies them to group semantics to track the crowd status and predict any potential accidents and disasters. For example, participant’s data such as names, ID numbers, group ID, destination locations, contacts, and necessary health information can be registered into the tracking bracelet. In case of an emergency, the data could be used to facilitate help by the public safety personnel. By using proposed algorithms, it can predict a few potential problems in the mobile crowd scenarios. It also detects speed among the moving groups as well as identify a possible collision by measuring the flow density. The proposed monitoring approach is explicitly designed for densely crowded environments. We have conducted various practical mobile crowd tests in both indoor and outdoor environments under different crowd conditions from low to high-density. The results show that by integrating the BLE data metrics, the system can identify the crowd density, the object group location, and the flow direction and speed in real-time.

The paper structured in the following. First, we overview some recent existing works. Second, we are explaining ICE-MoCha design including the approach’s design and scenarios. Next, we explain the methodologies of our experiments. Then, we review and evaluate the results. Lastly, we conclud our paper with final comments.

## 2. Related Work

Crowd monitoring and tracking topics are attracting the attention of researchers due to its importance for urbanizing cities. There have been several recent crowd management studies that address the issue of tracking the massive crowd using video cameras or wireless technologies. In this section, we review the most related works in both video surveillance and radio frequency RF-based tracking.

**Video surveillance** is one of the most common and traditional ways for safety and security monitoring. Several papers addressed the issue of crowd density risks and proposed videos surveillance to estimate the density or monitor the crowd [6,10]. However, video surveillance requires manual data analysis; it cannot respond in real-time. In addition, it is not accurate to track and estimate the high-density crowd using cameras because obstacles such as wall, tree and the human body can block the camera’s vision from capturing objects [11]. Head detection using cameras is another approach because the human head is the most visible part of the human body at a crowd from the camera’s tower. Shami et al. [12] proposed an algorithm that detects atpeople’s heads in a crowd for counting the density using convolutional neural network (CNN). However, the accuracy of capturing the human heads can be affected in case if some pedestrians have umbrellas or small persons blocked by large persons from the camera’s view. Bek et al. [4] proposed an approach to measure the crowd density flow for congestion risk assessment. The study used a single camera tracking example without taking into consideration that large crowds require more than one camera, so in case of multiple cameras, they may have different measurements in tracking the moving crowd for risk assessment [13]. Alahi et al. [13] proposed unsupervised technique learning to match multiple camera single-view in tracking pedestrians by estimating the distance between pair cameras. Matching the view of multiple cameras to track pedestrians is a great effort. However, their work did not take into account object navigating. For example, in the case of out-view objects during moving from one camera’s angle to another or in the case of obstacles blocking a camera’s view of an object. Therefore, due to the accuracy and capability limitation of the video surveillance approaches, it cannot manage crowd events alone.

**RF-based tracking**, such as Wi-Fi, is another method of crowd managing which has many papers proposing solutions for crowd density estimation and tracking [14,15,16]. The authors in [14] used coordinated indoor Wi-Fi routers to collect data between TX and RX and used an SVM model to train the data to count the number of people in a room. The study in [15] attempts to localize people at TT festival in Assen, Netherlands and improved accuracy by de-noising the collected Wi-Fi data. Both studies focus on limited crowd characteristics. Nunes et al. [16] analyzed MAC addresses and associated SSIDs to study the dynamism of tourists, but it is for a static posterior analysis rather than a real-time crowd management. Li et al. [17] proposed a framework to capture probe packets sent by smart-phones and use it to monitor crowd density in the indoors. Also, they used RSSI to indicate the closest sensor to smart-phones to collect data to reduce packet duplication. Our work is different where we have focused on tracking crowd mobility indoor and outdoor environments using multiple metrics such as RSSI, beacon count, and time-stamp. Also, our work uses bracelets instead of smart-phones due to data privacy issues. Patil et al. [18] suggested Wi-Fi to track the number of people at a massive event. They capture probe packets of attendees’ smart-phone Wi-Fi to estimate the size of crowd. Their work is focused on estimating the number of attendees, while our work focuses on tracking crowd mobility at the events. Unlike the above, we use BLE instead of Wi-Fi due to its low power consumption, low cost, availability, flexibility in size, and wearable-friendly. RFID has been one of the most common wireless technologies to identify and track objects with an active RFID tag. Yamin et al. [19] used GPS equipped RFID tags connected to a centralized database to track pilgrims. This method also applies to Al-Hashedi et al. who proposed using RFID connected to a data center to track pilgrims during Hajj [20] and Mitchell et al., who also mentioned the possibilities of using RFID along with smart-phones to track the pilgrims during Hajj [21]. In our work, we are using BLE instead of RFID because the RFID system cannot support any communication based intelligent monitoring approaches [22]. GPS is a satellite-based system that has been used for navigating and tracking objects in outdoor environments. Blanke et al. [23] suggested using GPS for tracking crowd in large scale areas, but GPS has limitation coverage for indoor environments event, in which it does not support our study in this paper. BLE is low power wireless technology that has been used to connect smart devices. There are a couple of papers used BLE in their proposed solutions. Basalamah et al. [24] used an active mode Bluetooth Low Energy (BLE) tag. The beacon messages are scanned by smart-phones (detectors). However, the active BLE tags consume the battery power quickly. It also increased the chance of overhead and packet collisions at dense events. In facts, this approach decreased the data accuracy since the people carried the smart-phones (detectors) within the crowd. In contrast, our approach takes a passive mode tag that improves data accuracy, scalability, and power consumption. Weppner et al. [25] used smart-phones to scan for other Bluetooth devices to estimate the crowd density. However, this work did not provide any additional intelligent measurements for managing the crowds. Also, their work mainly focused on assessing the static crowd density, while our work tackles a mobile crowd to handle the crowd mobility and safety [26]. Alessandrini et al. [27] used RSSI in Wi-Fi for localization in the crowd and to track the flow. We are using RSSI in BLE [28] as a tool for localizing objects. Several papers studied RSSI in BLE for indoor localization. Wu et al. [11] used the BLE RSSI captured by three signal sniffers to classify if people are in a queue during the crowd at indoors. Our approach is different because we used BLE RSSI from the respond beacons, then we used RSSI average and variation to detect the crowd density and mobility indoor and outdoor events.

## 3. ICE-MoCha Architecture

Mobile crowd management is one of the hardest tasks because predicting human behavior during a crowded event is extremely difficult. In ICE-MoCha, we study the feasibility of using BLE beacon signals from various BLE transmitters for tracking the mobile crowd status.

### 3.1. ICE-MoCha Layer

**Figure 3:**
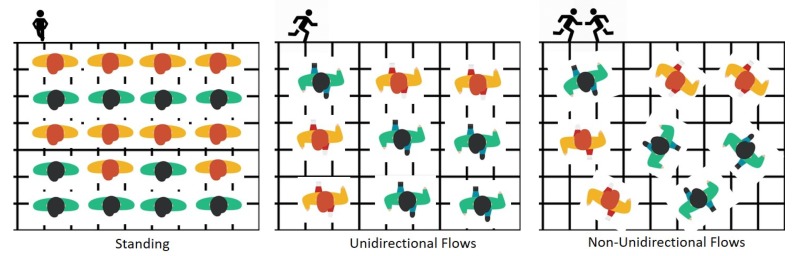
Crowd density illustration.

**Figure 4:**
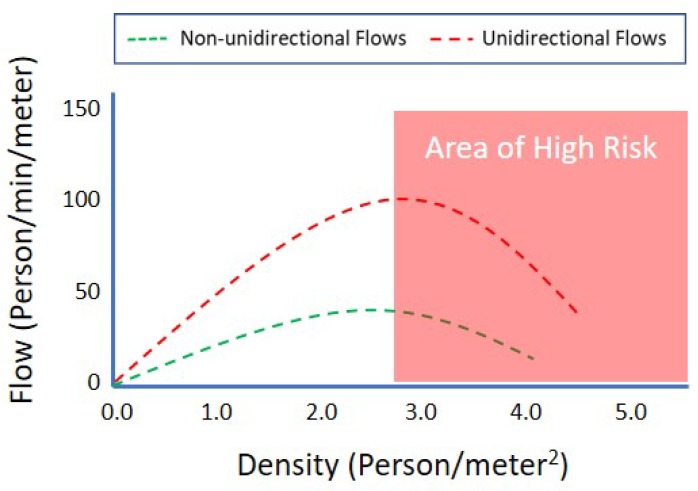
Mobile crowd density vs. flows.

According to the crowd safety and risk analysis [29], understanding the impact of crowd density (the number of people per square meter) for both a standing crowd and a mobile crowd is critical for managing crowd safety. For example, to assess the efficiency of crowd movement, a capacity of places, it needs to understand the relative risks of both standing crowd density and the moving crowd density. In some case, if a standing crowd becomes mobile and a unidirectional crowd becomes non-unidirectional, the planned capacity design fail. It can cause any unexpected disasters. Figure 3 illustrates the standing, unidirectional, and non-unidirectional density in people per square meter. At low densities (i.e., one person per square meter), the mobile crowd is free-flowing and stable, and the standing crowd is safe and comfortable. As illustrated in Figure 4, when the crowd density (the number of people per square meter) increases, the comfort level of the crowd decreases and flow speed starts to decrease as people cannot take full paces forward. After a saturation point, crowd mobility becomes constrained and accumulated, and the flow rates dramatically drop. For the crowd moving in the same direction, when density becomes more than three people per square meter, the flow speed starts to decrease, and when density becomes more than four people per square meter, the flow speed drops to become a high-risk crowd. However, when the crowd moves randomly in different directions, the flow speed decreases significantly, and the lower density and even at a density of 3 or 2.5 people becomes a high-risk crowd. In a low-density case, a collision can be avoided by stopping the flows. However, in a high-density case, when crowd force pushes people in the front forward, shock waves began to ripple through the tightly packed mass, and it causes a crush and crowd disasters. The crowd safety management should be able to predict the potential flow directions well before the crowd is getting into a high-crowd condition.

**Figure 5:**
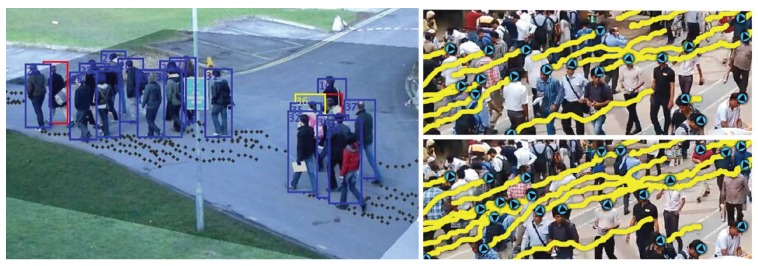
Smart video surveillance.

As shown in Figure 5 [7], using the advanced artificial intelligence (AI) technology and machine learning (ML) algorithms, the intelligent video surveillance enables us to detect and track multiple moving objects. However, it cannot scale to monitor various objects in a high-density crowd due to the limitation of visual processing. Also, video surveillance cannot follow the moving objects if obstacles or another human block them. It is hard to handle the hand-over from one camera angle to another [13]. Furthermore, the video surveillance alone cannot identify and predict particular crowd status such as group semantics. For example, it does not expose the group identity and location within the crowd and their moving direction and speed. Hence it alone lacks the capacity of providing an appropriate crowd safety management in real-time.

ICE-MoCha enhances the safety management method for the mobile crowd by harnessing a BLE signal data analytics layer over the existing video surveillance. Among the many crowd mobility characteristics, by using a BLE bracelet and BLE scanners, ICE-MoCha measures the beacon counts, the RSSI power, and its variation pattern. Although these metrics are used in various applications, their behavior in a high-density is not well known. By integrating the parameters and application-specific semantics over the video surveillance, ICE-MoCha can identify the crowd density, the object group location, and the flow direction and speed in both indoor and outdoor environments. ICE-MoCha also can predict any potential accidents and disasters. For example, participant’s data such as names, ID numbers, group ID, destination locations, contacts, and necessary health information can be registered into the tracking bracelet. By using group speed and direction detection algorithms, ICE-MoCha can predict a potential collision in various mobile crowd scenarios.

### 3.2. ICE-MoCha Design

ICE-MoCha uses a Bluetooth low energy (BLE) communication. As illustrated in Table 1, BLE is known to be more energy efficient than other wireless technologies such as classic Bluetooth and Wi-Fi [30]. The coverage range of BLE, over 100 m, is as good as others. It is enough to cover the densely populated crowd area. ICE-MoCha consists of the BLE tracker bracelets worn by a human, the BLE scanners, and the scanning algorithms. Each BLE tracker bracelet has a unique identification-ID to identify each bracelet.

#### 3.2.1. BLE Scanning Approaches

**Figure 6:**
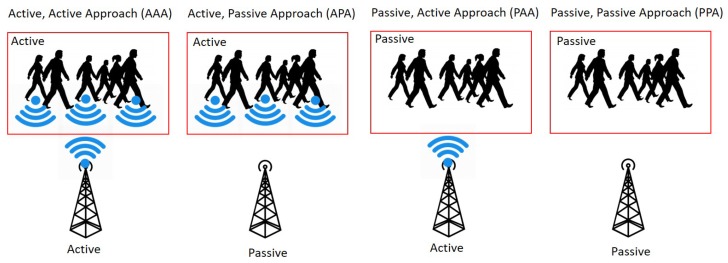
BLE Scanning Approaches.

In a wireless communication system, there are a couple of common messaging modes. In a passive mode, a node does not send any periodic message but scans incoming messages. In an active mode, a node periodically sends messages to indicate its existence. As illustrated in Figure 6, the combination of these two modes are used between the BLE scanners and the pedestrians’ bracelets (BLE trackers) to communicate the crowd states. A tuple of the BLE tracker mode and the BLE scanner mode approaches including an active-active approach (AAA), an active-passive approach (APA), a passive-active approach (PAA), and a passive-passive approach (PPA) were investigated to ensure efficiency in power usage, increase the scalability in message communications, and improve the accuracy in event detection.

- **An active/active approach (AAA)** has a better chance of capturing most of the pedestrians’ bracelets data because the BLE scanners point is sending a request and waiting for a response, in the same time the pedestrians’ bracelets are on active mode, sending requests, and waiting for responses. However, this will cause higher power consumption on both sides, which is one of our significant concerns in improving battery consumption. That does not only increase battery consumption, but it also increases the number of requests and responses causing overhead and increasing the probabilities of data collisions.
- **An active/passive approach (APA)** is a passive scanning approach for the BLE scanners. The pedestrians’ bracelets keep on sending their locations. The BLE scanners listen and respond upon receiving messages. The pedestrians’ bracelets are always active, the power consumption on the bracelet is one of the main concerns, and one of the primary research focus is to reduce the power consumption on the pedestrians’ bracelets. Also, the number of messages is proportional to the number of bracelets. Hence, for a densely populated environment, the approach may increase the message overhead and cause a high chance for message collisions.
- **A passive/active approach (PAA)** is a BLE scanner driven approach. The BLE scanners are sending polling or probing requests. The pedestrians’ bracelets are listening and responding upon receiving the request messages. As the pedestrians’ bracelets are passive, the power on the bracelet is efficiently utilized. Also, the number of messages is kept to a minimum as the bracelets are responding upon the requests. As the control is in the BLE scanner side, the responding messages from the bracelets can be efficiently controlled as well. The approach can decrease the message overhead and maintain a smaller chance of message collisions.
- **A Passive/Passive Approach (PPA)** does not perform any active probing. Both the BLE scanners and the pedestrians’ bracelets are on listening mode. Although this approach can save power usage, it does not provide any meaningful information about the moving groups. A possible option is if the control room can detect a low traffic situation (during the off-peak times) by using other methods such as CCTV.

In a passive BLE scanner mode, BLE scanner does not perform any periodic active probing by assuming that BLE tracker periodically sends beacon messages. However, if the BLE tracker bracelets sends beacon messages periodically, the power consumption on each bracelet is one of the primary concerns. Besides, the number of messages is proportional to the number of bracelets. Hence, in a densely populated crowd, the passive BLE scanner approach can significantly increase the message overhead and cause a high chance for message collisions. Meanwhile, in an active BLE scanner mode, a BLE scanner periodically sends polling or probing requests. The BLE tracker bracelets are listening and responding upon receiving a probe message. As the BLE tracker bracelets are in a listening mode without periodically sending beacon messages, it can maintain its power consumption efficiently. Besides, the number of beacon messages is kept to a minimum as the bracelets are responding only to the requests. As the control is in the BLE scanner side, it can adequately control the number and period of beacons according to the size and density of the crowd. The approach can decrease the message overhead and maintain a smaller chance of message collisions. ICE-MoCha uses a passive BLE tracker mode and an active BLE scanner mode approach (PAA). An ICE-MoCha probing message includes a sampling factor by indicating a replying pattern. For example, a BLE scanner specifies the BLE IDs for a few specific groups. Using this methodology reduces the probabilities of collisions. It also helps to decrease the power consumption of the BLE tracker bracelet because only the bracelet with the specified ID will reply in response to the polling messages.

#### 3.2.2. Crowd Detection Scenarios

**Figure 7:**
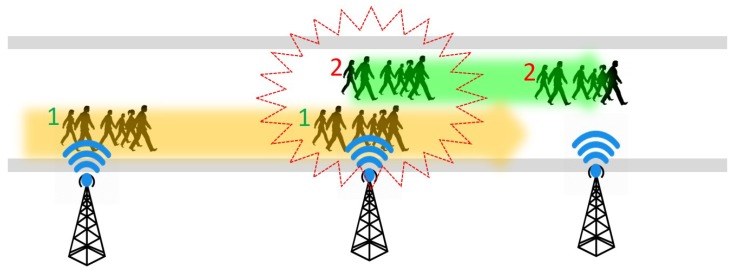
Group speed detection illustration.

**Figure 8:**
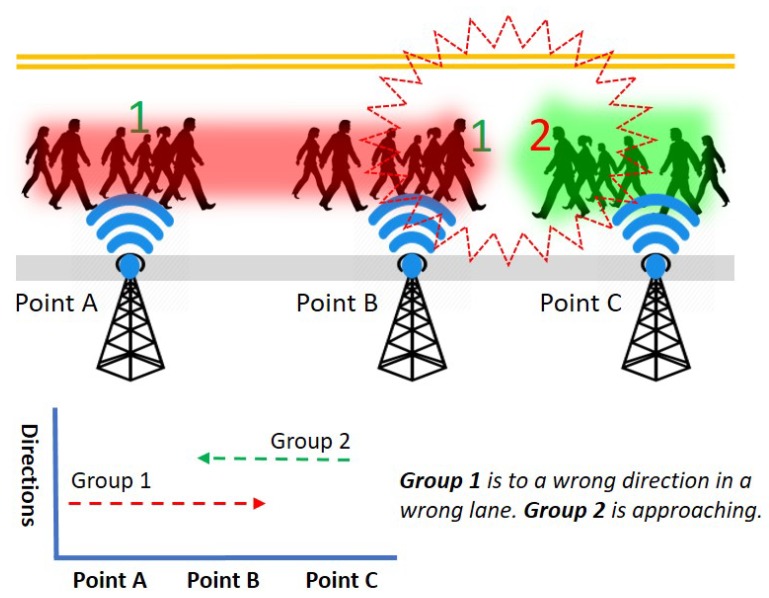
Wrong lane group detection via direction.

**Group speed and direction detection:** Even if the crowd is moving in the same direction, the moving speed can be different from one group to another. It may cause congestion and collision by the fast-moving groups. For example, Figure 7 illustrates the scenario where a couple of groups are walking in the same direction. However, a fast-moving group 1 in the back causes congestion by taking up the front group 2. ICE-MoCha can detect the moving speed of each group from the time-stamp and the distance from BLE scanners at point A to B. If the speed of each group can be detected earlier, the system could predict any potential collision. Assuming the average walking speed of a human is 3.1 mph (5 kph) [8], ICE-MoCha can identify the speed of each group and gives a warning to the fast-moving groups or members. Furthermore, if group 2 is moving slower than the average speed, the system can alert group 2 to speed up or give a slow down warning to group 1. The BLE scanners also coordinate to detect the movement direction of each group. By comparing the time-stamps of each groups’ passing position, ICE-MoCha can also detect the moving directions of each group. For example, Figure 8 illustrates that a group 2 is moving from the BLE scanner point A to point B. However, assuming that group 1 is supposed to move in an opposite direction according to the schedule, ICE-MoCha can identify a potential wrong direction of group 1 (or a temporary backward movement). The wrong movement shall be alerted to the group and other neighbor group as it may result in a collision with upcoming group 2.

**Figure 9:**
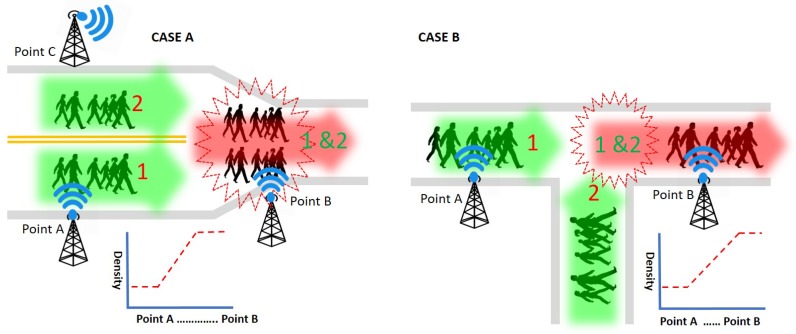
Lane merge detection via density.

**Figure 10:**
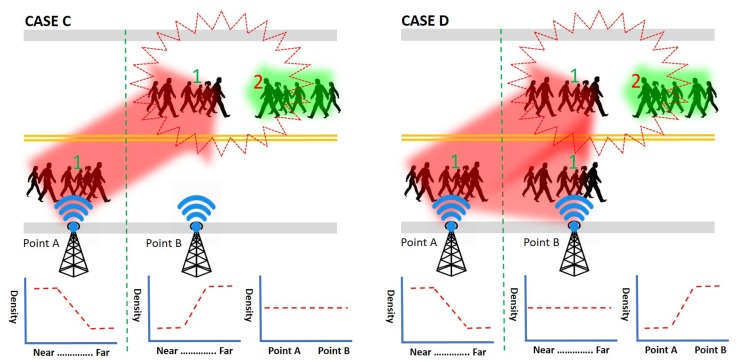
Wrong lane group detection via density.

**Group density detection:** ICE-MoCha can scan the density of each group within the monitoring range and shares the information with the neighbor scanners in real-time. In practice, a crowd collision (i.e., unexpected high density) often happens for various reasons. It can be due to the structure of the road as well as human errors. For example, as illustrated in Figure 9, two lanes are merged into one lane and various intersections mix flows into several directions. Also, shown in Figure 10, a group of people in a crowd may be veering off an opposite lane (when it is available) instead of using the slow and crowded path so that they can move faster. These cases can be identified by tracking the density changes on each point. Both Case A and Case B illustrate scenarios where groups 1 and 2 are merging into one lane due to the road design either merging lanes or intersection. BLE scanners can detect both cases by checking the density distribution changes on a scanning point B. The density at the BLE scanner point B becomes higher than the density at the BLE scanner point A. Case C illustrates a scenario where group 2 is, all of a sudden, changing its path in the middle of the road to veer off the opposite lane. Case D is a similar scenario, but group 2 takes both lanes. BLE scanners can detect Case C by checking the density distribution changes on a scanning point. At the BLE scanner point A, the density near the scanner is high, but the far side is low. However, in the BLE scanner point B, although the total density is the same, the density near the scanner becomes low and the far side changes to high. BLE scanners can also detect Case D by checking the density changes. At the BLE scanner point A, the density near the scanner is high, but the far side is low (like in Case C). However, the density at the BLE scanner point B becomes higher than the density at the BLE scanner point A, as well as the density near the scanner and the far side of the scanner, become comparable. The density distribution can be measured by multiple scanners using the RSSI power and beacon counts (shown in experimental results).

### 3.3. ICE-MoCha Framework Deployment

Although ICE-MoCha has been designed as an RF-based surveillance layer along with the existing video surveillance technologies, it can be deployed as a standalone safety management framework. ICE-MoCha framework consists of three main hardware components including a BLE transmitter tag, a BLE signal scanner, and an analytic server as well as various software components including parameter integration and application-specific semantics creation algorithms. Firstly, a BLE transmitter tag (i.e., a bracelet, sticker, necklace, shoe, etc.) is worn by the individual object to emit a simple RF signal with its a unique identification (ID). Secondly, a BLE signal scanner (i.e., an infrastructure placed nearby the event area) efficiently collects RF signals and relay them to a backend analytic server. Thirdly, an analytic server runs various algorithms to request, store, analyze data, create information to report alerts. Also, a BLE signal scanner runs an efficient signal collection algorithm instead of passively scanning the incoming RF signals. For example, to avoid any potential signal collision, a BLE scanner uses a sampling algorithm for its probe request. It can distribute the response patterns by specifying BLE IDs for a few specific groups for a probe period so that only the BLE transmitter tag with the specified ID will reply in response to the request. Among the many BLE signals, analytic server algorithms use beacon counts, the RSSI power, and its variation pattern parameters. By integrating those parameters and applying application-specific semantics, ICE-MoCha can identify the crowd density, the object group location, and the flow direction and speed. ICE-MoCha also can predict any potential accidents and disasters. For example, participant’s data such as names, ID numbers, group ID, destination locations, contacts, and necessary health information can be registered into the tracking bracelet. By using group speed and direction detection algorithms, ICE-MoCha can predict a potential collision in various mobile crowd scenarios.

ICE-MoCha deployment can be configured differently from one event scenario to another because each event has a different crowd characteristic. For instance, the type of crowd can be mainly static or mobile as well as the location and environments can be indoor or outdoor. Also, the monitoring duration can be occasional or regular. However, ICE-MoCha has initially been designed as a practical and field usable safety management framework for Hajj [31]. Hajj is the Muslim pilgrimage to Mecca that takes place in the last month of the Islamic calendar year. All Muslims are expected to make this pilgrimage at least once during their lifetime. Usually, in addition to the native Saudi Arabians who regularly go to Hajj, around two million Muslims from around the world gather in Mecca, Saudi Arabia. The actual Hajj period lasts for five days. During the period, people move among multiple locations including Mina, Mozdalifa, Arafat, and Jamarat (in addition to Mecca) that are a few miles apart from one another [31]. Some people walk while others use public transportation to get to the locations, but in most cases, they all end up walking because cars are not allowed during the most crowded times. Due to the lack of adequate crowd management methods, many serious incidents including crowds that collide and children who go missing are not uncommon sights [32]. Often, the identities of some of those casualties are not eventually discovered. Hence, they are buried in Saudi Arabia. Furthermore, in trying to identify the victims, the government of Saudi Arabia has to do extra work, which includes DNA testing that usually takes a very long time to process [33]. For example, sharing DNA samples with other countries isn’t an easy task for Saudi Arabia. Besides, for the many children who get lost during the Hajj, trying to find their group or family is an arduous manual process considering the huge mobile population at the event.

**Figure 11:**
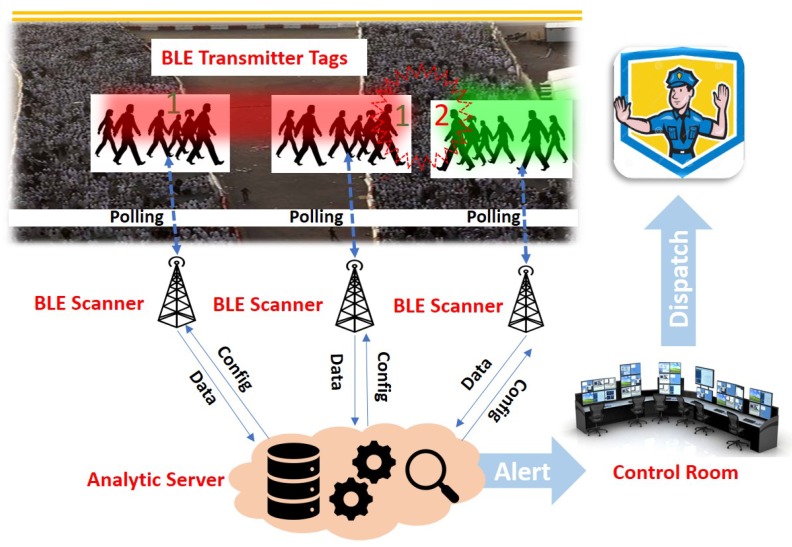
ICE-MoCha Deployment and Design.

After discussing with the government official of the Hajj event in Saudi Arabia for a potential deployment method, we identified the necessary background information. The type of crowd is mobile, the period of the event is five days, the environment is mostly outdoors, the crowds move in the form of groups, not all of the attendees carry smart-phones, and if they have them, they will use it for an emergency, and the age of pilgrimages vary from children to adults. Also, we identified that the pilgrims’ or attendees’ information such as names, ID numbers, camp numbers and locations, contacts, and necessary health information could be securely placed into the tracking bracelet during the registration process. In case of an emergency, the data could be retrieved by the public safety personnel to facilitate help such as looking for the group or camp for a lost child or identifying a specific casualty. According to the information we have acquired, we prototyped an ICE-MoCha framework for a Hajj event. We designed a disposable BLE transmitter that had a low cost (i.e., $1 to $2 for a BLE chip, battery lasted for ten days to make the total cost in a range from $3 to $10) with versatile form-factors in design and size (i.e., harnessed as a sticker, bracelet, necklace, or shoe). As illustrated in Figure 11, each BLE scanner periodically sends request messages to BLE transmitters with specified tag IDs to reply. Collecting beacon counts, the RSSI power, and its variation pattern parameters from the BLE transmitter tags, a BLE scanner conveys the data to an analytic server. By integrating those data, the analytic server applied the ICE-MoCha algorithms to characterize the crowd density, the object group location, and the flow direction and speed. In the case of any suspicious crowd movement such as using the wrong direction, moving too fast, etc., the server sent a detailed alert message to the situation control room to do the necessary action such as dispatching the local police officers and the emergency medical technicians (EMTs). For example, if one group was moving in the opposite direction on the road, the control room got a warning from the ICE-MoCha server. The control room can check the situation to notify police officers who are near to the group location to guide the flow in the correct direction. Besides, the information can also be used to predict any potential congestion and suspicious movements that may cause future disasters. The control room authorities can revise their safety management plans including additional infrastructure placement and policeman locations.

## 4. Evaluations

As ICE-MoCha is a mobile cyber-physical system in a densely populated environment, the communication feasibility issue should be evaluated in a real environment [4]. Hence, we verify how human object movements could affect the BLE signal in a crowded environment.

### 4.1. Experimental Setup

**Figure 12:**
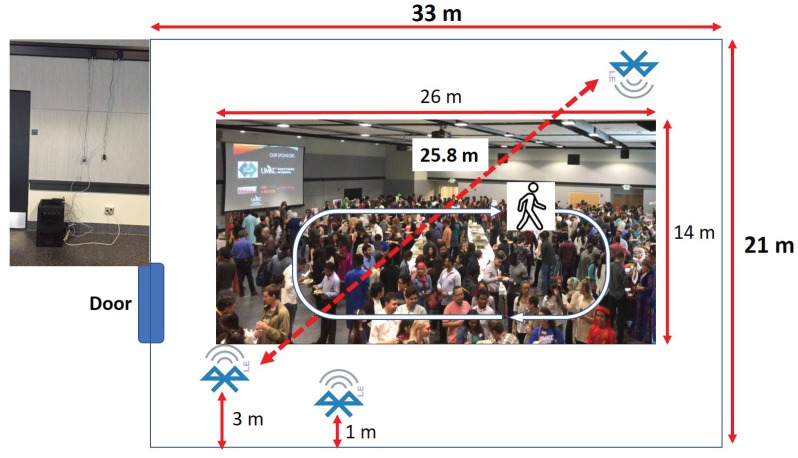
Culture night indoor experiment.

We conducted experiments at a couple of different settings. First, we experimented during one of the largest events at UMKC called “Culture Night”, where around 1300 people from different countries are presenting their cultures in a conference hall. The goal of this experiment was to study how significantly interference and crowd density could affect ICE-MoCha. As the conducted experiments were mainly for the feasibility analysis, we wanted to see if the BLE signal from a BLE transmitter tag can indicate any crowd status. For the purpose, only one BLE transmitter within the crowd was carrying a smart-phone with an application that advertises its BLE signal. Also, to explore multiple scenarios during the event to establish a better understanding of crowd status. As illustrated in Figure 12, a conference hall size was about 700 m^2^ (33 m * 21 m), and people area was about 370 m^2^ (26 m * 14 m). We posted a couple of BLE scanners on an opposite side placed at the height of 3 m. The distance between the two BLE scanners was about 25 m. We also put another BLE scanner placed at the height of 1 m next to the door. A moving human object walked around the hall in a circle.

**Figure 13:**
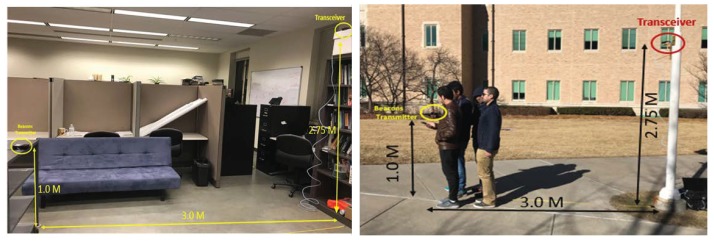
Indoor and outdoor RSSI experiment setup.

Second, we conducted RSSI experiments both indoors and outdoors. As shown in Figure 13, we placed a BLE transceiver at 2.75 m height and a BLE transmitter at 1 m height in 3 m apart. We put human interference from none to three or four people near the BLE transmitter. We set up the transceiver to scan for a minute each test, with a total of ten separate times for each test session.

**Figure 14:**
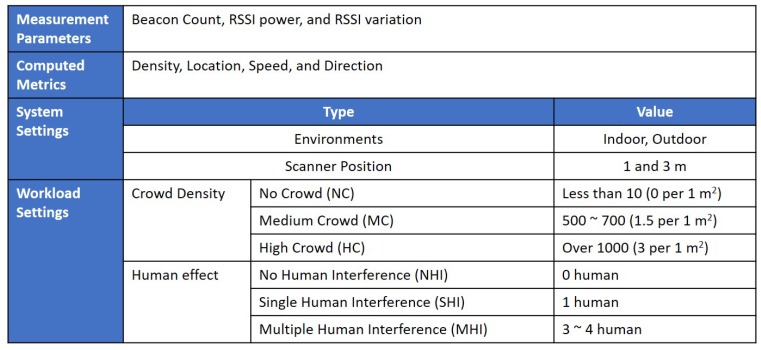
Experimental Settings.

As illustrated in Figure 14, we measured beacon count, RSSI power, and RSSI variation metrics to detect crowd density, location, speed, and direction. We tested in both indoor and outdoor environment with different scanner positions (1 m and 3 m) as the system settings. The workload parameter, in Figure 14, consists of a couple of sets. The crowd density parameters characterized in no crowd (NC), medium crowd (MC), and high crowd (HC). For example, given a 370 m^2^ human area of the hall, when there were 1000 people, it was about three people in 1 m^2^. We classified it as HC as it belonged to the high-risk case for the free moving environment. In the RSSI experiment, the human effect parameter consists of no human interference (NHI), single human interference (SHI), and multiple human interference (MHI). As the RSSI testing environment was at 3 m distance, a few human-objects can make a high crowd effect.

### 4.2. Beacon Count Tests

Counting beacons for a given time to find a population seems to be a straightforward approach. However, when there was a mass of crowds, the result may not be the same due to the collision and interference.

**Figure 15:**
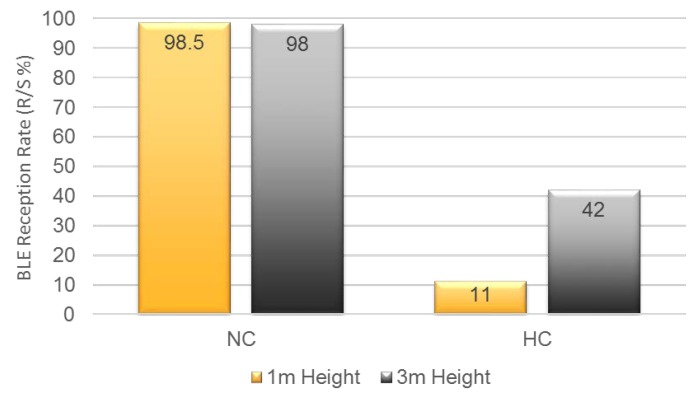
BLE reception vs. scanner height in crowd.

Figure 15 presents the results of the BLE reception rate (i.e., received (R) percentage of sent (S) beacons) for the different heights in both NC and HC environments. We setup the BLE transceiver heights for one meter (low) and three meters (high), respectively. In general, it shows that the higher crowd (HC) there were, the higher number of beacon messages were dropped. The result shows that the message-receiving ratio in HC environment was about 50% less than that in NC environment. Furthermore, the reception rate of the higher BLE detector (three meters) was about 31% higher than that of the lower BLE detector (one meter). In this result, we can see that the efficient BLE detector location (in height) is above every human height to avoid any signal absorption by the crowds.

**Figure 16:**
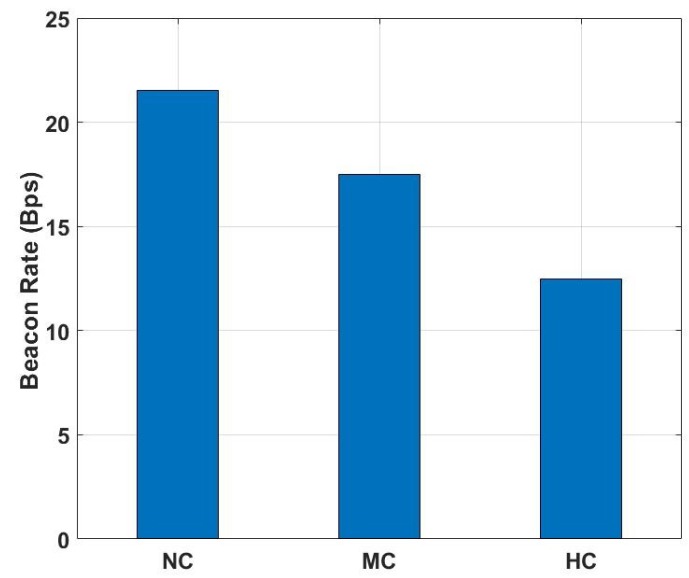
Beacon per second (Bps).

Figure 16 shows the average received beacons per second (Bps). For the experiment, we configured the BLE transmitter’s beacon advertising interval to 20 ms. Adding a random delay of 0–10 ms and a scan interval of 10 ms, a BLE scanner can get a beacon about every 40 ms, which means it received around 24–25 Bps. The results show that in the NC environment the received beacons are around 23 Bps while in the HC environment it decreased to 13 Bps. The results indicate that human object certainly had an impact on the received beacon count. The effect is proportional to the size of the crowd.

**Figure 17:**
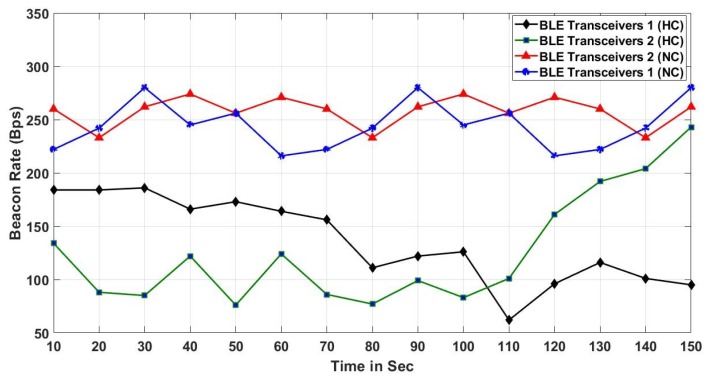
Beacon counts for BLE scanners.

Figure 17, compares the results of beacon counts on two separate BLE scanners (posted in an opposite side of a hall) for both NC and HC conditions while an object is moving in a circle as showing in Figure 12. The HC result exhibits a pattern that the beacon count increased when a moving object approached a specific BLE transceiver, while the beacon count decreased in the other BLE transceiver. In addition to exposing the proximity of the moving object, the result also infers the moving speed and direction (i.e., for two BLE transceivers, it will display approaching and going.) We observed that pattern became evident when the crowd density increased. Meanwhile, the NC result shows that both BLE transceivers received almost the same number of beacons regardless of a moving object location. As a result, it indicates that when the environment is NC, object tracking is not possible. However, receiving a similar amount of beacons on both BLE scanners and the beacon counts are more than HC condition, we can observe that the crowd condition is at low risk.

### 4.3. RSSI Tests

**Figure 18:**
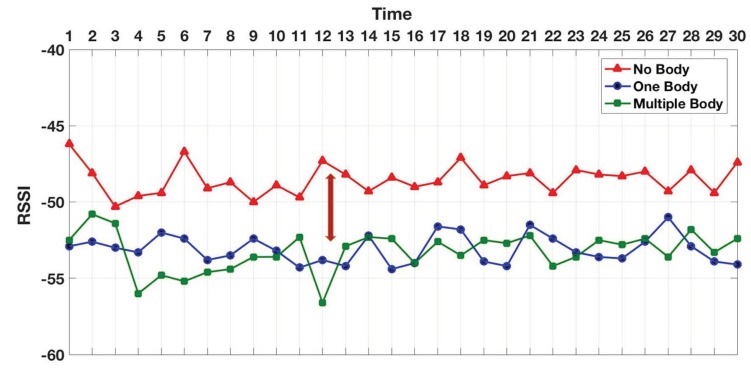
Indoor average RSSI.

**Figure 19:**
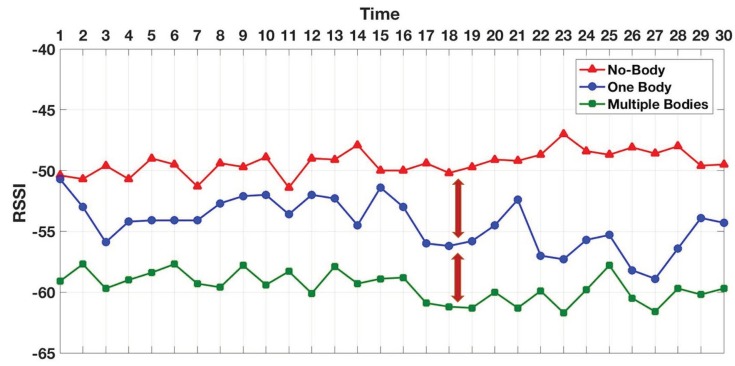
Outdoor average RSSI.

**Figure 20:**
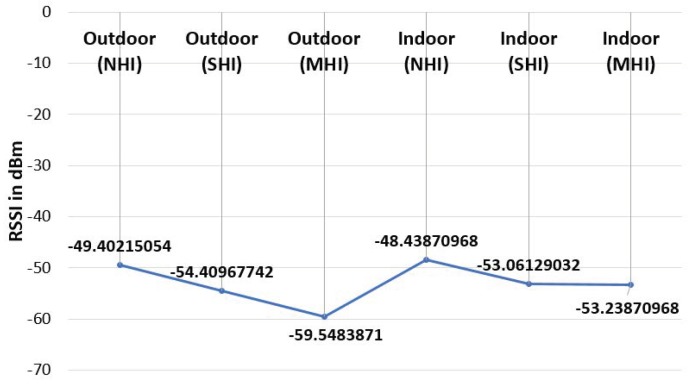
Average RSSI comparison.

In addition to beacon counting, we further measured a couple of BLE RSSI metrics including the RSSI power and variation. In both indoor and outdoor environments, we tested under three workload conditions including NHI, SHI, and MHI. The indoor test results in Figure 18 show that the average RSSI power in NHI was stronger than both SHI and MHI, while there was no significant difference in the signal power average between the SHI and MHI. On the other hand, the outdoor average RSSI power results in Figure 19 show that NHI received stronger signal RSSI power than SHI, and SHI had stronger signal RSSI power than MHI. As shown in Figure 20, the total indoor RSSI power of both SHI and MHI is the same, while the total outdoor shows each NHI, SHI, and MHI RSSI powers show a clear difference.

In summary, both indoor and outdoor results indicate that they can identify any human interference (i.e., NHI vs. non-NHI). However, the indoor case cannot discern the density level difference (i.e., SHI vs. MHI). Unlike the indoor case, the outdoor case can distinguish the different density levels. It indicates that the RSSI power metric alone can identify a coarse level of the human interference, especially the indoor case.

**Figure 21:**
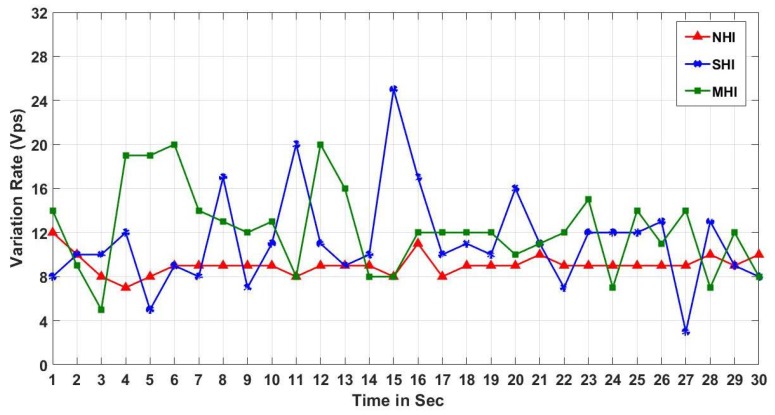
Indoor RSSI variation.

**Figure 22:**
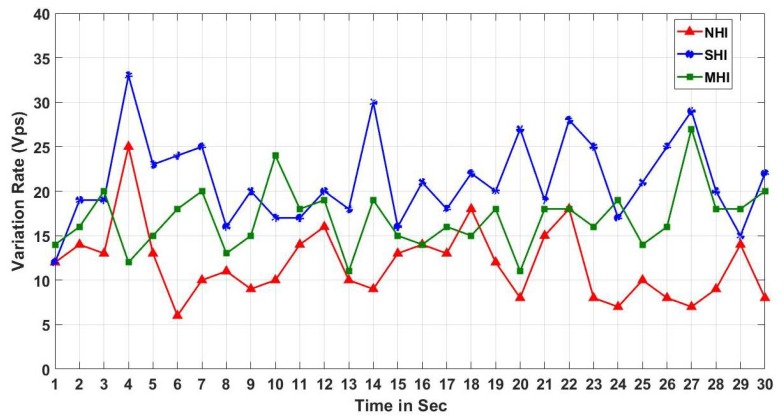
Outdoor RSSI variation.

**Figure 23:**
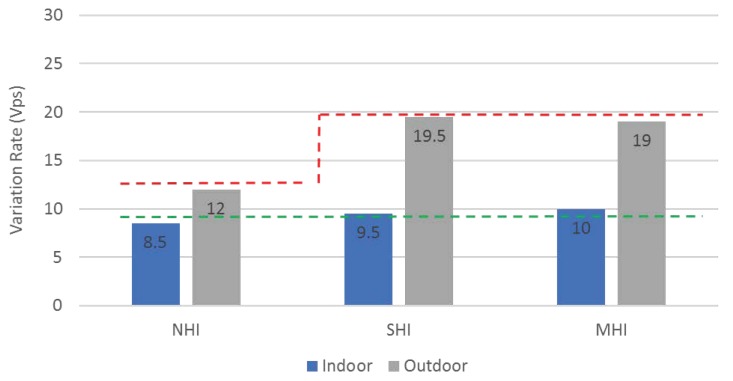
RSSI variation Comparison (Indoor vs. Outdoor).

We test a variation of the received RSSI power. We picked the maximum, and minimum RSSI power values among the beacons received in a second (Vps), and used the difference as a variation value. The indoor RSSI variation test results in Figure 21 show that the RSSI variation in NHI is stable while the RSSI variation in other non-NHI (i.e., SHI and MHI) is randomly fluctuating. On the other hand, the outdoor RSSI variation test results in Figure 22 show that the RSSI variation results in all workloads (NHI, SHI, and MHI) unstably vibrating. As shown in Figure 23, the average indoor RSSI variation values are stable in all workloads from 8.5 to 10 Vps. Although the average values were similar, the variation values in both SHI and MHI are fluctuated while NHI variation was stable. Meanwhile, the average outdoor RSSI variation values were much higher than the indoor variation average values and are also different between NHI and non-NHI. The non-NHI values were similar and higher than the NHI value. In summary, the indoor results indicate that they can identify any human interference (i.e., NHI vs. non-NHI). However, the outdoor case cannot discern any density. Hence, it indicates that the RSSI variation metric alone can identify a coarse level of indoor human interference. For example, it can be used to check if there is a person in the room or not, but it not possible to notice the crowd density.

### 4.4. Discussion

The primary purpose of this study is to improve crowd safety management method through real-time radio frequency (RF) to predict and prevent potential disasters. Our approach focused on characterizing and analyzing the crowd mobility in speed, direction, and density through BLE beacon count and RSSI power and variation. In summary, the findings from the experimental results include the following: the BLE beacon count approach can be used to detect a location, direction and the speed of an object during the crowd by coordinating multiple scanners. The RSSI power average can be used to identify human interference outdoors, while RSSI variation can check any human intervention indoors, but it cannot evaluate the density. Therefore, by integrating those metrics, ICE-MoCha can identify the flow direction and speed, and the crowd density and object group location.

## 5. Conclusions

One of the critical services in smart cities is the safety management of urban communities. However, it is very challenging to predict crowd clashes in real-time among the mobile crowds for preventing any potential disaster. In this work, we designed, implemented, and tested ICE-MoCha that enhances crowd mobility characterization through real-time BLE data analytics. The proposed ICE-MoCha can enhance or complement video surveillance based approaches and enables crowd management in a real-time and scalable manner using effective and efficient BLE signal analysis.

Among the crowd mobility characteristics, it identifies the crowd density, the object group location, and the flow direction and speed by analyzing BLE beacon counts, the radio strength signals received (RSSI) power, and its variation pattern. Addressing the scalability and capability issues of the smart video surveillance by tightly integrating BLE signal analytics, our work translates the signals into group semantics to track the crowd status and predict any potential accidents and disasters.

We have conducted various practical mobile crowd experiments in both indoor and outdoor environments under different crowd movement scenarios. We demonstrated the feasibility that our approach can effectively detect the direction, the location, the speed, and the density of the mobile crowd in real-time. We believe it sheds lights on future crowd safety management systems where more sophisticated crowd management functionalities could be enabled by augmenting with other approaches in a complementary manner.

## Figures and Tables

**Table 1:** RF transmission approaches.

Protocol	Range	Mobility	Deployment
BLE	≥100 m	≤5 Mph	Ubiquitous, Low power usage, low association time
WiFi	≥100 m	≤5 Mph	Ubiquitous, Low power usage, high association time
Cellular	≥10 Km	≥60 Mph	Ubiquitous, Low power usage, high association time
